# Protocol for processing multivariate time-series electronic health records of COVID-19 patients

**DOI:** 10.1016/j.xpro.2025.103669

**Published:** 2025-03-05

**Authors:** Zixiang Wang, Yinghao Zhu, Dehao Sui, Tianlong Wang, Yuntao Zhang, Yasha Wang, Chengwei Pan, Junyi Gao, Liantao Ma, Ling Wang, Xiaoyun Zhang

**Affiliations:** 1 Peking University School and Hospital of Stomatology, Beijing, China; 2 Peking University, Beijing, China; 3 Health Data Research, London, UK; 4 University of Edinburgh, Edinburgh, Scotland, UK; 5 Key Laboratory of High Confidence Software Technology (Peking University), Ministry of Education, Beijing, China; 6 Affiliated Xuzhou Municipal Hospital of Xuzhou Medical University, Xuzhou, Jiangsu, China; 7 Beihang University, Beijing, China

**Keywords:** Bioinformatics, Health Sciences, Computer sciences

## Abstract

The lack of standardized techniques for processing complex health data from COVID-19 patients hinders the development of accurate predictive models in healthcare. To address this, we present a protocol for utilizing real-world multivariate time-series electronic health records of COVID-19 patients. We describe steps for covering the necessary setup, data standardization, and formatting. We then provide detailed instructions for creating datasets and for training and evaluating AI models designed to predict two key outcomes: in-hospital mortality and length of stay.

For complete details on the use and execution of this protocol, please refer to Gao et al.^1^

## Highlights

• Steps for standardizing multivariate time-series EHR data format of COVID-19 patients
• Instructions for processing EHR data of COVID-19 patients for training AI models
• Guidance on training and evaluating AI models through tailored pipelines

## Abstract (editor highlights)

Publisher’s note: Undertaking any experimental protocol requires adherence to local institutional guidelines for laboratory safety and ethics.

## Graphical abstract

**Figure:**
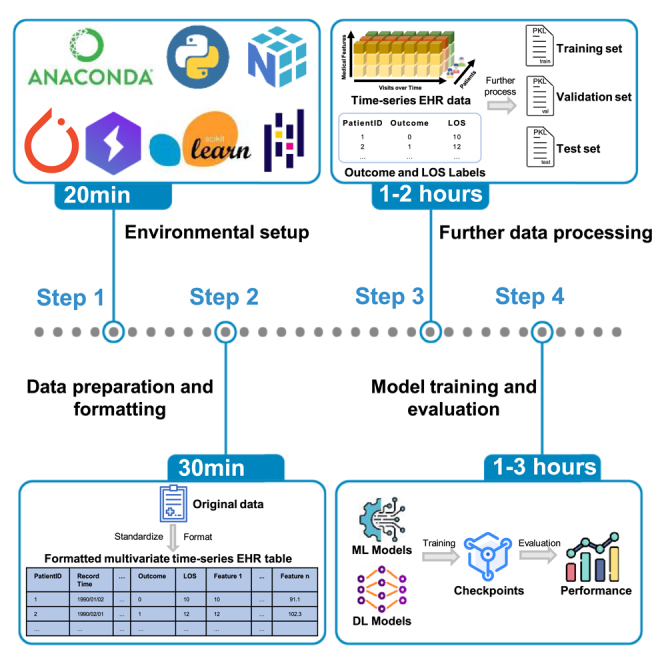

## Before you begin

The global healthcare landscape continues to face significant challenges from COVID-19, despite the emergence of less virulent variants. The virus’s enhanced transmissibility drives periodic case surges that disproportionately impact intensive care units (ICUs), highlighting a critical gap in our capacity to manage healthcare resources effectively.^2^ This ongoing pressure underscores the urgent need for accurate prognostic tools to improve patient outcomes and optimize resource allocation in the evolving post-pandemic era.

Artificial intelligence (AI) models have demonstrated remarkable potential in predicting patient outcomes, enabling clinical interventions and contributing to adequate treatment.^1^^,^^3^ However, the development and application of these AI models are dependent upon high-quality, large-scale data.^4^ A major obstacle is the lack of standard techniques for processing raw patient data from ICUs. This multivariate time-series electronic health record (EHR) data is complex, consisting of multiple clinical indicators (such as laboratory tests, vital signs, medications, and procedures) recorded across multiple patient visits over time. The absence of standardized methodologies for processing this raw clinical data presents a significant barrier to the widespread adoption of AI-driven solutions in clinical practice. To bridge this critical gap, we introduce a streamlined data processing protocol specifically designed for EHR data from COVID-19 ICU patients. This protocol aims to create a systematic approach to data harmonization and model development, thereby providing a standardized pipeline for enhancing patient outcomes and improving ICU efficiency both for the current pandemic and potential future public emergencies.

### Institutional permissions

The EHR dataset used in this study is publicly accessible and de-identified to safeguard patient privacy.^3^ We strictly followed their data usage policies throughout this study.

## Key resources table

**Table:** 

REAGENT or RESOURCE	SOURCE	IDENTIFIER
**Deposited data**		

Original data for COVID-19 patients in intensive care unit	Tongji Hospital	https://github.com/HAIRLAB/Pre_Surv_COVID_19
Source code	Present study	Zenodo: https://doi.org/10.5281/zenodo.14785998

**Software and algorithms**		

Anaconda	Anaconda>=Anaconda3-2024.06-1-Linux-x86_64	https://www.anaconda.com/
Python	Python>=3.11.10	https://www.python.org/
Pytorch	Torch>=2.5.1	https://pytorch.org/
Lightning	Pytorch-lightning>=2.4.0	https://lightning.ai/pytorch-lightning
Pandas	Pandas>=2.2.2	https://pandas.pydata.org/
NumPy	Numpy>=1.26.4	https://numpy.org/
Scikit-learn	Scikit-learn>=1.5.2	https://scikit-learn.org/stable/

**Other**		

Ubuntu	Canonical	Ubuntu 22.04.2 LTS
CPU	Intel	Intel(R) Xeon(R) Silver 4210R CPU @ 2.40GHz
GPU	NVIDIA	GeForce RTX 3090

***Note:*** We primarily utilize Ubuntu to execute the code for this study, leveraging both Intel CPUs and NVIDIA GPUs. Our code is also compatible with other operating systems, including Windows and macOS, as well as other CPU architectures (i.e., x86, ARM, etc.). GPU acceleration is not necessarily required for our project implementation.

## Step-by-step method details

### Environmental setup

**Timing: 20 min**

Here, we describe the installation scripts for required software environment and packages.

1. Download the project from Zenodo: https://doi.org/10.5281/zenodo.14785998 and unzip the file to obtain source code files.
2. Download Anaconda from https://repo.anaconda.com/archive and install it.
  >wget https://repo.anaconda.com/archive/Anaconda3-2024.06-1-Linux-x86_64.sh
  >bash Anaconda3-2024.06-1-Linux-x86_64.sh
  a. Execute the first command to download the installer of Anaconda of the specific version.
  b. Launch the installation process by executing the subsequent command.
    ***Note:*** During the installation, follow the prompts and press “ENTER” to select all the default options. For the two prompts below, input “yes”:
    Do you accept the license terms? [yes|no]
    Do you wish to update your shell profile to automatically initialize conda?
    This will activate conda on startup and change the command prompt when activated.
    If you’d prefer that conda’s base environment not be activated on startup,
    run the following command when conda is activated:
    conda config --set auto_activate_base false.
    You can undo this by running conda init --reverse $SHELL? [yes|no]
    ***Note:*** We recommend using the latest version of Anaconda. One can refer to the official Anaconda website for the latest updates.
3. Create an experimental environment.
  >conda create -n covid python=3.11
  >conda activate covid
  a. Execute the first command to create a new Conda environment named “covid” with Python version 3.11.
    ***Note:*** During the process, input “y” for the prompt below.
    The following NEW packages will be INSTALLED:
    … …
    Proceed ([y]/n)?
  b. Execute the second command to activate the newly created “covid” environment, making it your current working environment.
4. Install required software packages.

***Note:*** We provide a *“requirements.txt”* file containing all required libraries packages. One can run this command in the directory *“Fieldry-DataProcessCovid19-078cef8”* to install these packages at one time.

>pip install -r requirements.txt

**CRITICAL:** Verify that all required software packages listed in the key resources table are correctly downloaded and installed by importing them in Conda’s Python environment. If the environment setup fails, see (troubleshooting 1).

### Data preparation and formatting

**Timing: 30 min**

Here, we standardize the original data of COVID-19 patients obtained from medical institutions into a multivariate time-series EHR data table, and then format the table.

5. Download the raw data file “time_series_375_prerpocess_en.xlsx” from https://github.com/HAIRLAB/Pre_Surv_COVID_19/tree/master/data or from Yan et al.^3^’s paper’s Supplementary Information section.

***Note:*** One can directly download the raw data file by running the following command.

>wget -L

https://raw.github.com/HAIRLAB/Pre_Surv_COVID_19/master/data/time_series_375_prerpocess_en.xlsx

***Note:*** The raw data format is shown in Table 1. Each row in the EHR data table represents the observed clinical measurements for each patient at each ICU record. Please ensure compliance with its associated license terms and conditions when utilizing this dataset.

**CRITICAL:** The Python code for steps 6 and 7 is provided in the *“standardize_preprocess.py”*, and one can run the following command in the directory *“Fieldry-DataProcessCovid19-078cef8”* to achieve the processing steps. Before using the code script, please put the raw data file in the directory *“datasets/tjh/raw”*.

6. Standardize EHR data for each patient at each ICU record in the raw data table into a multivariate time-series EHR data table, as shown in Table 2.

***Note:*** In the multivariate time-series EHR data table, the first column represents a unique identifier for COVID-19 patients, labeled as “PatientID”. The second column represents the time point of the record. The third and fourth columns represent the time points of admission and discharge for patients in intensive care, respectively. The fifth column represents the clinical outcome, 0 and 1 indicating survival and death at the end of the ICU period respectively. The columns from the sixth to the last represent demographic information and laboratory test results at the nth time point.

7. Format the multivariate time-series EHR data table to obtain a formatted multivariate time-series EHR data table, as shown in Table 3.
  a. Format data values.
    i. Convert Gender values to binary: Assign a value of 1 for male patients and a value of 0 for female patients.
    ii. Keep the year/month/day (Y/M/D) format for RecordTime, AdmissionTime, and DischargeTime.
  b. Clean missing data.
    i. Ensure that all records are complete for PatientID, RecordTime, AdmissionTime and DischargeTime. Remove any records with missing data in these columns to ensure consistency.
    ii. Drop columns whose values are all missing or all the same.
  c. Merge records by date.
    i. Group the data by PatientID and RecordTime to combine entries for the same patient on the same day.
    ii. Calculate the mean of numeric values within the grouped data to generate a single record per patient per day.
      ***Note:*** In actual clinical practice, not all laboratory tests are conducted at every ICU time-point. We merge records on a daily basis to reduce the overall rate of missing data within the dataset. In cases where other datasets exhibit more frequent recording of laboratory features, it’s feasible to merge records at finer temporal resolutions, such as by the hour, minute, or even second, etc.
  d. Calculate Length of Stay (LOS).
    i. Compute LOS in days by subtracting RecordTime from DischargeTime.
    ii. Set any negative LOS values to 0 to correct potential errors.
      **CRITICAL:** The steps required to standardize and format the multivariate time-series EHR data table may vary depending on the dataset (troubleshooting 2).

**Table 1:** Raw EHR data table

Patient_ID	Re_DATE	age	Gender	Admission time	Discharge time	Outcome	Feature 1	…
–	–	–	–	–	–	–	–	…
–	–	–	–	–	–	–	–	…
–	–	–	–	–	–	–	–	…

**Table 2:** Multivariate time-series EHR data table

PatientID	Record time	Admission time	Discharge time	Outcome	Feature 1	…	Feature m
1	1990/01/02 01:09:00	1990/01/01 22:12:00	1990/01/11 12:40:00	0	1.0	…	91.1
1	1990/01/02 02:19:00	1990/01/01 22:12:00	1990/01/11 12:40:00	0	NaN	…	NaN
…	…	…	…	…	…	…	…

**Table 3:** Formatted multivariate time-series EHR data table

PatientID	Record time	Admission time	Discharge time	Outcome	LOS	Feature 1	…
1	1990/01/02	1990/01/01	1990/01/11	0	10	1.0	…
2	1990/02/01	1990/02/01	1990/02/13	1	12	2.1	…
…	…	…	…	…	…	…	…

### Further data processing

**Timing: 1–2 h**

Here, we further process the formatted multivariate time-series EHR data table, to construct a dataset designed to train AI models.

**Figure 1:**
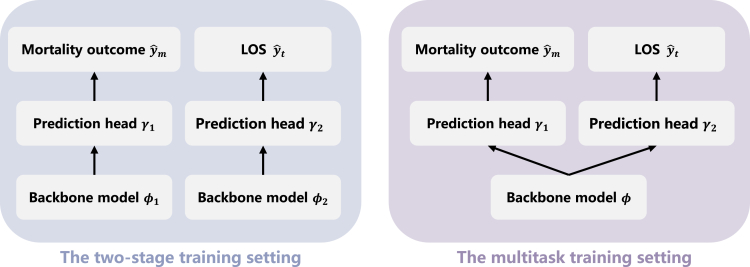
Illustrations of two training approaches

**Figure 2:**
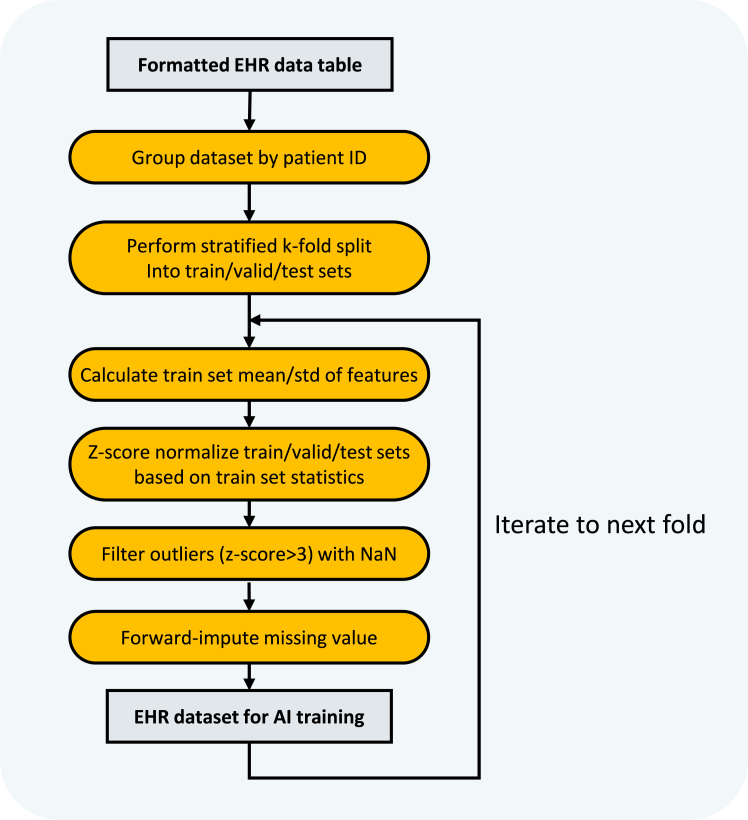
Processing the formatted multivariate time-series EHR data table for training AI models

8. Define the tasks and labels.
  a. Define the tasks:
    $${\hat{y}}_{m,t},{\hat{y}}_{l,t}=model\left(R_{t}\right),t=1,2,\ldots ,T$$
    ***Note:*** Given the formatted multivariate time-series EHR data R of a patient, we train AI models to predict two tasks at each ICU point: the binary outcome classification task and the LOS regression task. In practice, assuming a patient has T ICU records, given the patient’s first to t-th ICU records $R_{t}$, the model predicts the patient’s clinical outcome and remaining days after the t-th ICU point to the end of the ICU stay.
  b. Define the labels.
    ***Note:*** For the binary outcome classification task, the label $y_{m,t}\;\epsilon \;\left\{0,1\right\}$ signifies whether the patient will succumb by the end of the ICU stay, where 1 for mortality and 0 for survival. For the LOS regression task, the label $y_{l,t}\geq 0$ indicating the remaining days to the end of the ICU stay.
    ***Note:*** For both labels, the closer the predicted $\hat{y}$ is to the label y, the higher the accuracy of our AI models.
    **CRITICAL:** We design two training approaches for these two tasks, as depicted in Figure 1. First is an end-to-end multitask learning strategy where a single model backbone with two prediction heads jointly predicts both mortality and LOS. The second is two-stage training strategy where separate models predict outcome and LOS independently. We provide options for implementing both strategies. For a more detailed comparison of these two approaches, please refer to Gao et al.^1^
    **CRITICAL:** The Python code for step 9 is provided in the *“further_process.py”*, and one can run the following command in the directory *“Fieldry-DataProcessCovid19-078cef8”* to achieve the processing steps.
9. Construct data for training and evaluating AI models based on the formatted multivariate time-series EHR data table, as depicted in Figure 2.
  a. Group the dataset by PatientID, and perform the stratified k-fold cross-validation strategy to split the dataset into training set, validation set and test set with a (k-2):1:1 split ratio.
    ***Note:*** In the stratified cross-validation strategy, the dataset is randomly divided into k subsets, with each part taking turns as the test set while the remaining parts are used for training and validation. The split is performed by stratifying based on the Outcome label, ensuring that the distribution of outcomes is consistent across all subsets. This step is recommended for minimizing the randomness of the test-sample selection process.
    ***Note:*** The number of folds and the split ratios are customizable. In our study, k is set to 10 with an 8:1:1 ratio for training, validation, and test sets, respectively. One can customize settings according to your preferences and requirements. For example, a 7:1:2 ratio can also be employed for these sets. Based on our previous experiments on this dataset, the split ratio will not significantly impact the results.
  b. Calculate the mean values, median values and standard deviation of all features using the data in 5%–95% quantile range in the training set to avoid the outliers’ influence.
  c. Construct the dictionary $los_{info}.pkl$ indicating the information of LOSs.
    i. Retain the first LOS value of all patients, filter the new LOS values in 5%–95% quantile range and calculate the mean value of the filtered data.
    ii. Construct the dictionary with five key-value pairs, including “los_mean”, “los_median”, “los_std”, “large_los” and “threshold”.
      ***Note:*** The values of “los_mean”, “los_median” and “los_std” are obtained from the previous step b. The value of “large_los” is the 95% quantile of the filtered data. The value of “threshold” is the half of the mean value of the filtered data. These values are used to calculate metrics.
  d. Normalize the data using z-score normalization:
    $$z=\frac{\left(x-\mu \right)}{\sigma +eps}$$
    ***Note:*** The normalization is applied to all demographic features, laboratory tests features and LOSs. x is the original value, μ and σ is the mean value and standard deviation of the data in the training set respectively. If all the features are the same, the standard deviation σ will be zero, which makes the denominator zero. To prevent division by zero, a small value eps (e.g., 1 × 10^−12^) is added to the denominator.
  e. Filter outliers after normalization and replace them with “NaN” (Not a Number) values to avoid the outliers’ influence.
    ***Note:*** The outlier value $z^{*}$ is defined as $|z^{*}|>3$ in our study, which effectively filters out the vast majority of extreme values. For stricter outlier filtering, the threshold can be set higher than 3.
  f. Impute the missing data using the following imputation strategies.
    i. Use the Last Observation Carried Forward (LOCF) method to impute the missing value if a feature has a missing value and a previous observation exists.
      ***Note:*** LOCF method is an imputation strategy where the missing value is replaced with the last observation value.
    ii. Use the median value calculated from the training set to impute the missing value if a feature has a missing value and no previous observation exists.
      ***Note:*** Alternative imputation strategies for missing values can be used. For instance, impute all missing values with the mean values, median values or other statistical measures calculated from the training set.
  g. Construct the lists $train_{x}.pkl$, $val_{x}.pkl$ and $test_{x}.pkl$ with dimension (N,T,D).
    ***Note:***N represents the number of patients, T denotes the number of visits, and D is the number of medical features. T is different for every individual patient.
  h. Construct the lists $train_{y}.pkl$, $val_{y}.pkl$ and $test_{y}.pkl$ with dimension (N,T,2) for all visits based on the patient’s clinical outcome and LOS, in accordance with the label definition.
    ***Note:***N represents the number of patients, T denotes the number of visits and 2 is the number of target labels. T is different for every individual patient.
    ***Note:*** The steps from b to h are conducted separately for every fold in the cross-validation strategy setting.

### Model training and evaluation

**Timing: 1–3 h**

Here, we train and evaluate AI models using the dataset constructed.

10. Configure all the hyperparameters for training and evaluating process via the *“configs/config.py”* file.
  a. Configure hyperparameters for machine learning models.
    ***Note:*** The hyperparameters for machine learning models include: (1) “model” specifying the machine learning model; (2) “dataset” specifying the dataset; (3) “task” specifying the prediction task; (4) “seed” specifying the random seed for training process; (5) “batch_size” specifying the size of batch for every epoch during the training process; (6) “learning_rate” specifying the learning rate during the training process; (7) “main_metric” specifying the main metric to be monitored for improvement; (8) “max_depth” specifying the maximum depth of the tree for machine learning models, such as DecisionTree; (9) “n_estimators” specifying the number of boosting rounds for machine learning models, such as Catboost and XGBoost.
  b. Configure hyperparameters for deep learning models.
    ***Note:*** The hyperparameters for deep learning models include: (1) “model” specifying the deep learning model; (2) “dataset” specifying the dataset; (3) “task” specifying the prediction task; (4) “seed” specifying the random seed for training process; (5) “batch_size” specifying the size of batch for every epoch during the training process; (6) “learning_rate” specifying the learning rate during the training process; (7) “epochs” specifying running epochs during the training process; (8) “patience” specifying the number of epochs with no improvement after which training process will be stopped; (9) “main_metric” specifying the main metric to be monitored for improvement; (10) “demo_dim” specifying the number of demographic information in the dataset; (11) “lab_dim” specifying the number of laboratory values in the dataset; (12) “hidden_dim” specifying the dimension of hidden layers; (13) “output_dim” specifying the dimension of the output layer, 2 for multitask prediction (mortality and LOS) or 1 for single-task prediction (either mortality or LOS); (14) “accelerator” specifying the accelerator for training process; (15) “devices” specifying the devices to use if using “GPU” as the accelerator.
    ***Note:*** The “task” parameter for deep learning models determines the training strategy. Setting it to “multitask” enables the multitask training strategy where a single model backbone with two MLP prediction heads jointly predicts both mortality and LOS. Setting it to either “outcome” or “los” trains a single-task model that predicts only mortality or LOS respectively.
    ***Note:*** We provide the default values of all needed configuration hyperparameters used in this study in the “configs/config.py” file. For hyperparameter tuning, one may establish a search range based on these defaults (typically between 0.1 and 10 times the default values) and employ grid search methodology to determine optimal hyperparameter settings.
    **CRITICAL:** Ensure that the “demo_dim” and “lab_dim” hyperparameters correspond to the dataset (troubleshooting 3).
11. Run the *“train_evaluate.py”* file to train and evaluate AI models.
  a. Load the training/validation/test dataset files.
    i. For machine learning models: Stack the data x and y in list format to form a tensor with dimension $\left(\sum_{i=1}^{N}T_{i},D\right)$ for training set, validation set and test set respectively.
      ***Note:*** Machine learning models typically struggle to handle time-series data. After the data is stacked, machine learning models only take the information from the current time-point to perform prediction tasks at each ICU point.
    ii. For deep learning models: Do padding operations on the data x and y in list format to form a tensor with dimension $\left(N,\max\;\left(\left\{T_{i}|i=1,2,\ldots ,N\right\}\right),D\right)$ for training set, validation set and test set respectively.
      ***Note:***N represents the number of patients, $T_{i}$ denotes the number of visits of the i-th patient, and D is the number of medical features.
  b. Call the *trainer.fit()* function to train AI models (troubleshooting 4) (troubleshooting 5).
    ***Note:*** This process consists of two phases: training and validation. For every epoch, the model learns from data in the training set and updates its weight, and then the model is evaluated on the validation set. The process monitors the “main metric”, selects one model with the best performance on the validation set, and stops if there is no improvement after several epochs. The logging and checkpoint files during the training process are saved in the *“logs/train”* folder.
  c. Call the *trainer.test()* function to evaluate AI models.
    ***Note:*** The model with the best performance on the validation set is finally evaluated on the test set. The performance is saved to *“results/performance.csv”*.

## Expected outcomes

After the training process, the following files are generated in the *“logs/train”* folder: (1) *hparams.yaml*: This file contains all configuration hyperparameters. (2) *metrics.csv*: This file records model performance metrics for each epoch during training and validation. (3) *checkpoints/best.ckpt*: This file stores the model weights that achieved optimal performance on the validation set.

After the evaluation process, the final evaluation results on the test set are saved in *“results/performance.csv”*.

## Quantification and statistical analysis

**Figure 3:**
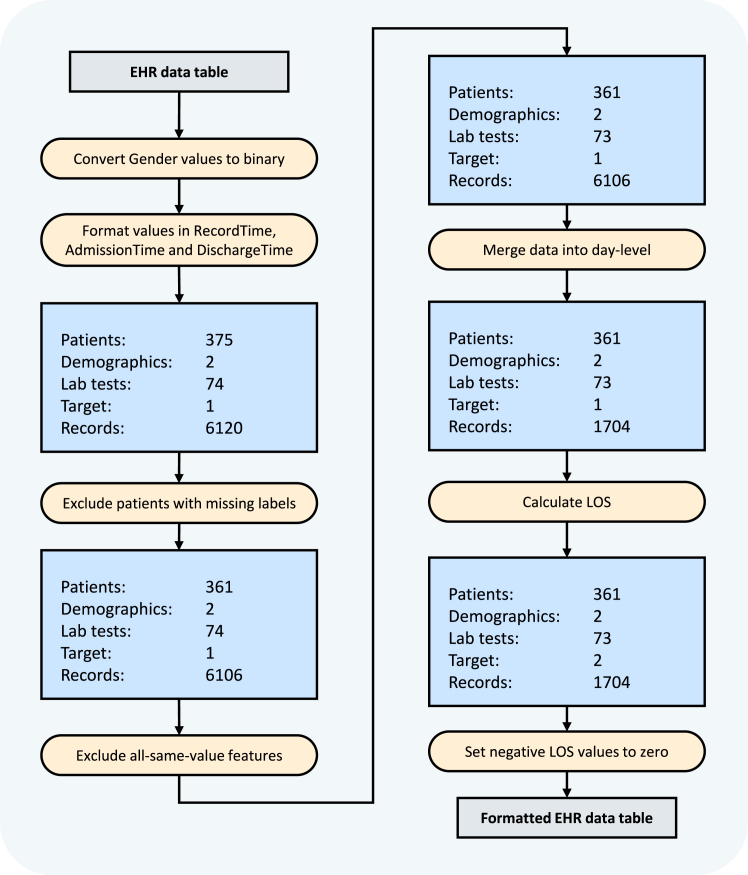
Inclusion and exclusion criteria for multivariate time-series EHR data Figures reprinted with permission from Gao et al., 2024.^2^

The details of the formatting steps for the multivariate time-series EHR data and the criteria for data inclusion and exclusion are demonstrated in Figure 3.

The evaluation metrics used are listed as follows:

1. For the binary outcome classification task: AUPRC, AUROC, Accuracy, F1, and EScore.
2. For the LOS regression task: MAE, MSE, and R2.
3. For multitask: OSMAE, and all metrics above.

For the complete information about metrics, please refer to Gao et al.^1^

## Limitations

This protocol outlines a comprehensive pipeline for processing and analyzing multivariate time-series EHR data of COVID-19 patients in ICUs. However, the protocol is predicated on the dataset from a single source, and tailored processing steps are needed for datasets from different medical institutions. Moreover, the performance of AI models is highly sensitive to hyperparameters such as hidden layer dimensions and learning rates, and may be unstable due to the lack of systematic hyperparameter optimization in our method.

## Troubleshooting

### Problem 1

Installation of Anaconda or required Python software packages fails (related to Step 2 and Step 4).

### Potential solution

Attempt to install Anaconda using the official installation guide available at https://www.anaconda.com/download. Then, proceed to reinstall the software packages as described in Step 4.

### Problem 2

Variations in data provided by different medical institutions may necessitate adjustments to formatting steps (related to Step 7).

### Potential solution

Despite slight differences in medical features, adhere to the core principles of formatting steps for the multivariate time-series EHR data table, which include.

• Ensure the presence of unique patient identifiers and ICU record timestamps in the multivariate time-series EHR data table.
• Format data values and clean error records.
• Define and calculate the label using columns in the multivariate time-series EHR data table if the target label does not exist.

### Problem 3

During the training process, errors may occur due to incorrect “demo_dim” or “lab_dim” configuration hyperparameters (related to Step 10b).

Traceback (most recent call last):

File "train_evaluate.py", line 80, in <module>

perf, outputs = run_func(config)

File "train_evaluate.py", line 63, in run_dl_experiment

trainer.fit(pipeline, dm)

… …

File "torch/nn/modules/rnn.py", line 892, in forward

self.check_forward_args(input, hx, batch_sizes)

File "torch/nn/modules/rnn.py", line 821, in check_forward_args

self.check_input(input, batch_sizes)

File "torch/nn/modules/rnn.py", line 240, in check_input

raise RuntimeError(

RuntimeError: input.size(-1) must be equal to input_size. Expected 77, got 75

### Potential solution

Verify the correctness of the two hyperparameters and attempt to train models again.

### Problem 4

During the training process, errors may arise if the calculated loss or parameter gradients become “NaN” values (related to Step 11b).

### Potential solution

This error occurs when the input data of the training process contains “NaN” values. Check the imputation step (Step 9f) and verify that there are no “Nan” values in the data after this step.

### Problem 5

During the training process, insufficient GPU memory may be encountered due to the large scale of model parameters (related to Step 11b).

### Potential solution

Use the command below to monitor the usage and free memory of the GPU during training.

Then, reduce memory usage by decreasing the “batch_size” parameter or select an alternative device if multiple GPU devices are available.

>nvidia-smi

## Resource availability

### Lead contact

Junyi Gao is the lead contact of this study and can be reached through e-mail: junyi.gao@ed.ac.uk.

### Technical contact

Zixiang Wang is the technical contact of this study and can be reached through e-mail: wangzx@stu.pku.edu.cn.

### Materials availability

This study did not generate new unique reagents.

### Data and code availability

The raw data used in this study can be accessed at https://github.com/HAIRLAB/Pre_Surv_COVID_19/tree/master/data. The code and processed data have been deposited to Zenodo: https://doi.org/10.5281/zenodo.14785998.

## Acknowledgments

This work was supported by the 10.13039/100014717National Natural Science Foundation of China (62402017 and 82241052), 10.13039/501100004826Beijing Natural Science Foundation (no. L244063), Xuzhou Scientific Technological Projects (KC23143), and Peking University Medicine plus X Pilot Program-Key Technologies R&D Project (2024YXXLHGG007). J.G. acknowledges the receipt of studentship awards from the 10.13039/501100023699Health Data Research UK-The Alan Turing Institute Wellcome PhD Programme in Health Data Science (grant 218529/Z/19/Z).

## Author contributions

Conceptualization, J.G., L.M., L.W., and X.Z.; methodology, Y. Zhu, J.G., L.M., and X.Z.; software, Z.W. and Y. Zhu; validation, Y.W. and C.P.; data curation, Y.W. and L.W.; writing – original draft, Z.W., D.S., and Y. Zhang; writing – review and editing, Z.W., Y. Zhu, and T.W.; supervision, Y.W., C.P., and J.G.

## Declaration of interests

The authors declare no competing interests.
